# Integrated Protein–Lipid Digestion Profiles of Human Milk, Infant Formulas, and Dairy Ingredients Under Infant In Vitro Digestion

**DOI:** 10.3390/foods15172999

**Published:** 2026-08-26

**Authors:** Bingyu Chen, Qi Qi, Xuteng Wang, Xinyi Zhang, Xuchun Zhu, Chao Yan, Huiwen Guan, Rong Jin, Yu An, Jie Yang, Fei Xu, Hongzhi Liu

**Affiliations:** 1 Key Laboratory of Geriatric Nutrition and Health, School of Food and Health, Beijing Technology and Business University, Beijing 100048, China; bychen@btbu.edu.cn (B.C.);; 2 Feihe Research Institute, Heilongjiang Feihe Dairy Co., Ltd., Beijing 100015, China

**Keywords:** infant formula, human milk, in vitro digestion, protein digestion, fatty acid profiling

## Abstract

Human milk (HM) represents the physiological reference for infant digestion, whereas infant formulas (IFs) differ in protein composition and lipid architecture. This study compared the gastric and intestinal digestion of HM, five commercial IFs, whole milk powder (WMP), and protein ingredients using a standardized infant in vitro model. Protein hydrolysis was assessed through digestibility and residual subunit patterns, and lipid digestion was evaluated through free fatty acid release and fatty acid profiling. Gastric digestion revealed pronounced differences driven by intrinsic structure and processing, whereas intestinal digestion reduced these disparities, although distinct subunit patterns persisted. Lipid analyses showed clear source-dependent separation before digestion and partial convergence during intestinal lipolysis. Several IFs containing structured lipid and vegetable-oil systems showed intestinal fatty acid or free fatty acid profiles closer to HM than WMP, whereas samples with stronger bovine-fat signatures remained more distinct. Overall, the results demonstrate that digestion-product patterns provide mechanistic insight into structure-driven digestion behavior in complex dairy-based matrices, beyond compositional comparison alone.

## 1. Introduction

Human milk (HM) is widely recognized as the gold standard for infant nutrition, providing an optimal balance of proteins and lipids with high digestibility and multiple bioactive functions [1,2,3,4]. Meanwhile, international and national regulations define strict ranges for the energy, protein, and fat contents of infant formulas (IFs) [5,6,7], resulting in relatively limited variation in macronutrient levels across commercial products. Beyond meeting basic nutritional requirements, modern IFs employ a range of targeted protein- and lipid-level formulation strategies to better approximate the structural and digestive characteristics of human milk (HM). On the protein side, α-lactalbumin (α-LA), the predominant whey protein in HM, is often enriched to optimize amino acid composition and to influence heat-induced unfolding and aggregation, thereby modulating gastric coagulation behavior [8,9]. Recent cellular studies further suggest that adjusting the relative levels of α-LA and β-casein (β-CN) may affect trans-epithelial transport of amino acids and minerals, indicating that protein subunit composition may influence digestive behavior and absorption-related pathways [8]. Lactoferrin (LF), an important HM whey glycoprotein with metal-binding and immunomodulatory structural features, is also incorporated into some formulas to adjust the whey-protein profile [10,11,12]. Hydrolyzed whey protein (HWP), composed of low-molecular-weight peptides with higher solubility, is considered to have enhanced digestive utilization and represents another used class of protein ingredients [13,14]. On the lipid side, structured triacylglycerols enriched in sn-2 palmitate (e.g., OPO) reproduce the stereospecific positioning of palmitic acid in HM fat, thereby influencing calcium-soap formation and fatty acid release patterns [15,16,17]. Differences in fat-globule size and interfacial composition may further contribute to divergent lipolysis pathways during digestion [15,18].

Variations in raw material properties and formulation-induced matrix structures can therefore lead to distinct digestive behaviors. Several studies have provided important insights by examining individual digestion indicators, such as protein hydrolysis, subunit degradation, and fatty acid release [19,20,21,22,23,24,25]. However, fewer investigations have evaluated the overall similarity of digestion trajectories within a unified framework. These studies demonstrate the value of standardized infant digestion models; however, comparison of HM and IFs within such models does not by itself fully explain how protein and lipid digestion behaviors are coordinated across ingredient and product levels. Protein-related endpoints and lipid-related endpoints are often interpreted separately, making it difficult to determine whether HM-like digestion behavior is mainly associated with compositional similarity, ingredient-level protein matrices, lipid-source design, or digestion-induced structural changes. For proteins, intestinal-phase residual subunit patterns may differ markedly despite comparable overall hydrolysis, reflecting differences in ingredient composition and processing conditions. For lipids, comprehensive assessments of whether the evolving fatty acid profiles of different IFs converge toward those of HM during digestion are still scarce. Overall, integrated evaluations of digestive-product pattern similarity are lacking, particularly studies that simultaneously include protein ingredients, finished IFs with diverse design principles, dairy controls, and HM.

To address these gaps, this study was designed as an integrated protein–lipid digestion comparison and employed a standardized static infant digestion model encompassing three categories of samples: (i) protein ingredients commonly used in IF manufacturing (e.g., α-LA, HWP, whey protein concentrate 80(WPC-80)); (ii) commercial dairy products, including five IFs formulated with different nutritional and structural strategies, together with whole milk powder (WMP) as a dairy reference; and (iii) HM as the biological reference. Protein digestion was assessed through apparent digestibility and intestinal residual subunit patterns, whereas lipid digestion was evaluated by quantifying free fatty acid (FFA) release and changes in fatty acid composition. Cluster analysis and module-wise similarity assessments were applied to compare how digestion-product profiles differed from HM across protein matrix, particle behavior, protein digestion, FFA release, and fatty acid composition.

## 2. Materials and Methods

### 2.1. Sample Information

Eleven samples were included in this study and categorized into three groups: (i) protein ingredients; (ii) commercial dairy products; and (iii) HM.

The protein ingredient group comprised four dairy-derived proteins commonly used in IF manufacturing: α-lactalbumin powder (α-LAP), HWP, WPC-80, and an α-LAP/HWP mixture (α-LAP + HWP). These ingredients were included to evaluate protein digestion characteristics at the ingredient level.

The commercial dairy product group included five IFs formulated with different nutritional and structural strategies, together with WMP as a dairy reference. The five IFs were selected as commercially available products with different formulation features, including differences in protein and lipid design, rather than to represent the full market or rank commercial brands. To avoid commercial bias, the five IFs were anonymized using sample codes (ZR, QC, FF, LY, MS). All powdered samples (protein ingredients, IFs, and WMP) were obtained as unopened commercial batches and stored under recommended conditions prior to analysis.

HM consisted of milk collected from five healthy lactating donors at 4–5 months postpartum. Approximately 200 mL of milk was collected from each donor using commercial breast milk storage containers (Pigeon, Chonburi, Thailand). Samples were immediately frozen after expression and stored at −20 °C to preserve native protein and lipid structures. All experiments were performed within one week of sample collection. Before digestion, the samples were thawed once at 4 °C, pooled in equal volumes, and immediately used for digestion to obtain a representative HM reference sample. The pooled HM sample was used as one biological reference matrix, and independent digestion experiments were performed using separately prepared aliquots of this pooled sample (*n* = 3). All donors provided written informed consent before sample collection. The study protocol was reviewed and approved by the Ethics Committee of Beijing Technology and Business University (Approval No. 137, approved on 14 June 2025). HM collection and use were conducted in accordance with the approved ethical guidelines.

### 2.2. In Vitro Simulated Gastrointestinal Digestion

Gastrointestinal digestion was conducted following the infant static in vitro digestion model described in the INFOGEST protocol and further adapted for full-term infants according to Ménard et al. and Zou et al., with minor adjustments to accommodate the characteristics of the tested samples [26,27]. Because the tested samples were liquid or reconstituted liquid milk systems, the oral phase was omitted, and the model included gastric and intestinal phases.

Powdered IFs were reconstituted at the manufacturer’s recommended concentrations, whereas HM was used without dilution. Thus, the commercial milk systems were compared under ready-to-feed or practical-use conditions. This approach was intended to reflect the way these products are prepared and consumed in practice, rather than to impose identical protein or lipid loads across all milk systems. Protein ingredients (α-LAP, HWP, WPC-80, and the α-LAP/HWP mixture) were prepared at formula-equivalent protein concentrations comparable to those of the reconstituted IFs, allowing comparison of ingredient-level protein digestion behavior under comparable protein loading. The initial protein, lipid, dry matter, pH, and estimated casein-to-whey ratio of the samples are provided in Table S1.

Simulated gastric fluid (SGF) was prepared following the infant digestion conditions described by Ménard et al. and Zou et al., with adaptations for the current study [26,27]. SGF contained 94 mM NaCl and 13 mM KCl and was adjusted to pH 5.3 with 1 mol/L HCl. The SGF contained porcine pepsin (P6887, Sigma-Aldrich, St. Louis, MO, USA) and gastric lipase from Rhizopus oryzae (62305, Sigma-Aldrich, St. Louis, MO, USA). In the original full-term infant static model reported by Ménard et al., rabbit gastric extract was used to provide gastric lipase activity, whereas Rhizopus oryzae lipase has been used as a practical substitute in modified infant in vitro digestion studies when rabbit gastric extract was not available [27,28,29]. The final activities in the gastric digestion mixture were 268 U/mL for pepsin and 19 U/mL for gastric lipase. Pre-warmed samples were mixed with SGF at a milk-to-gastric secretion ratio of 63:37 (*v*/*v*) and incubated at 37 °C for 60 min under gentle agitation at 50 rpm [29]. At the end of the gastric phase, the digestion was neutralized to pH 7.0 with 1 mol/L NaOH to stop gastric enzyme activity before intestinal digestion.

Simulated intestinal fluid (SIF) was prepared according to infant duodenal conditions reported by Ménard et al. and Zou et al. [27,29]. SIF contained 164 mM NaCl, 10 mM KCl, and 85 mM NaHCO_3_ and was adjusted to pH 7.0 before use. CaCl_2_ was added separately to reach 3 mM in the intestinal fluid. The SIF contained 3.1 mM bile salts (S30875, Shanghai Yuanye Bio-Technology Co., Ltd., Shanghai, China, total cholate hydrate content, 74.80%) and porcine pancreatin (P1750, Sigma-Aldrich, St. Louis, MO, USA). Pancreatin was added to provide final intestinal activities of 90 U/mL lipase and 16 U/mL trypsin. The neutralized gastric digestion was mixed with SIF to obtain a final meal:gastric secretion:intestinal secretion ratio of 39:23:38 (*v*/*v*/*v*), and the pH was adjusted to 6.6 after combination. The intestinal digestion proceeded for 60 min at 37 °C with controlled agitation at 50 rpm [29]. At the end of digestion, specific enzyme inhibitors were added before freezing to stop further proteolysis and lipolysis. Gastric proteolysis was inhibited using Pepstatin A. In intestinal digesta, proteolysis was inhibited using Pefabloc SC (4-(2-aminoethyl) benzenesulfonyl fluoride hydrochloride), and lipolysis was inhibited using 4-bromophenylboronic acid. The treated samples were stored at −20 °C until analysis [24,26].

### 2.3. Particle Size Distribution

The particle size distribution of the digestive samples was measured using a laser diffraction particle size analyzer (Mastersizer 3000, Malvern Instruments Ltd., Malvern, UK). Prior to analysis, samples were gently mixed and diluted tenfold with deionized water to maintain the obscuration of 7–10% to minimize multiple scattering [30]. The refractive index of fat droplets and the dispersant were set at 1.458 and 1.33, respectively, and the absorption index was set at 0.001. Each sample was measured in triplicate, and the average value was used for analysis. The particle size distribution data were obtained using the instrument software version 5.01.

### 2.4. Macroscopic Structural Observation

Macroscopic structural changes were evaluated through visual observation. After digestion, samples were transferred into transparent glass vials and stored at 4 °C for 0, 12, and 48 h to monitor visible phase separation and gastric curd formation. Images were captured at each time point using a digital camera (Nikon D7500, Nikon Corporation, Tokyo, Japan) under standardized lighting and distance conditions to ensure reproducible visual assessment.

### 2.5. Determination of Apparent Protein Digestibility

Protein digestion was evaluated as apparent protein digestibility based on the decrease in the residual trichloroacetic acid (TCA)-precipitable protein fraction. Briefly, 5 mL of sample was mixed with an equal volume of 24% (*w*/*v*) TCA, giving a final TCA concentration of 12% (*w*/*v*). The mixture was kept on ice for 10 min and then centrifuged at 10,000 r/min for 20 min. The supernatant was discarded, and the precipitate was washed with 6.7 mL of ice-cold ether/ethanol mixture (1:1, *v*/*v*), followed by centrifugation at 10,000 r/min for 10 min, and the supernatant was removed [31,32].

The protein concentration in the dissolved TCA precipitate from undigested samples and gastrointestinal digesta was determined using a Bicinchoninic Acid Protein Assay Kit (PC0020, Beijing Solarbio Science & Technology Co., Ltd., Beijing, China) following the manufacturer’s instructions. Samples from the gastric and intestinal phases were diluted appropriately to ensure that all measurements fell within the linear range of the standard curve. After mixing the diluted samples with the BCA working reagent, the reaction mixture was incubated at 37 °C for 30 min. Absorbance was measured at 562 nm using a microplate reader, and protein concentrations were calculated based on a standard curve generated from bovine serum albumin standards supplied with the kit.

Enzyme blanks containing the corresponding digestive fluids without sample were processed using the same procedure and subtracted from the measured values. Dilution factors introduced during digestion and sample preparation were included. The corrected residual TCA-precipitable protein concentration was calculated as:

Corrected residual protein = (Measured residual protein − Enzyme blank) × DF

Apparent protein digestibility was calculated as:

Apparent protein digestibility (%) = [(C_0_ − C_res_)/C_0_] × 100

where C_0_ is the initial protein concentration before digestion, C_res_ is the corrected residual TCA-precipitable protein concentration after digestion, and DF is the dilution factor introduced during digestion and sample preparation. Because TCA precipitation mainly recovers residual intact proteins and larger or less soluble peptides, the obtained values were interpreted as apparent protein digestibility rather than direct degree of hydrolysis or amino acid release.

### 2.6. Protein Profiles (SDS-PAGE)

Protein profiles of undigested samples and gastrointestinal digesta were analyzed using SDS-PAGE. A 2× SDS–PAGE sample buffer (20 mM Tris-HCl, pH 7.2, containing 2% *v/v* 2-mercaptoethanol, 10 g/L SDS, and 80 mg/L bromophenol blue) was freshly prepared. Liquid samples were mixed with the 2× buffer at a 1:1 ratio. For powdered samples and pellet-like digestion, a 1× sample buffer—prepared by diluting the 2× buffer with deionized water—was added at a 1:1 ratio to ensure complete solubilization.

All mixtures were heated at 95 °C for 5 min, cooled to room temperature, and centrifuged briefly to remove insoluble material. The supernatants were loaded onto 12% polyacrylamide gels together with a pre-stained protein ladder. Electrophoresis was performed at 220 V for 40 min. Following separation, gels were stained with Coomassie Brilliant Blue and destained until clear background was obtained. The resulting protein bands were used to assess residual subunit patterns across different samples and digestion stages.

### 2.7. Fatty Acid Composition Analysis

Fatty acid composition was determined from the lipid fraction extracted from undigested samples and digesta after acid-catalyzed methylation. Approximately 300 mg of sample was mixed with 2 mL of 5 percent sulfuric acid–methanol solution in a sealed glass tube, followed by the addition of 300 μL methyl acetate and 10 μL C17:0 FAME (5 mg/mL) as an internal standard. The mixture was vortexed, sealed under nitrogen, and heated in a 95 °C water bath for 1.5 h. After cooling to room temperature, 2 mL of 0.9 percent NaCl solution and 2 mL diethyl ether were added. The upper organic layer was collected after centrifugation (5000 rpm, 5 min). The extraction was repeated twice, and the combined organic phases were evaporated under a nitrogen stream. The residue was dissolved in 0.1 mL n-hexane prior to GC analysis. GC analysis was performed using an Agilent 7890A gas chromatograph (Agilent Technologies, Santa Clara, CA, USA) equipped with a flame ionization detector (FID) and a DB-FastFAME column (30 m × 0.25 mm × 0.25 μm; Agilent Technologies, Santa Clara, CA, USA). The injector temperature was 250 °C with a split ratio of 20:1. The oven program was set as follows: 80 °C for 1 min, increased to 165 °C at 40 °C/min and then to 230 °C at 4 °C/min, and held for 4 min. Detection was performed using FID at 260 °C. This method determined total fatty acid composition, including fatty acids derived from esterified lipids and FFAs, and was therefore not interpreted as a direct measure of FFA release.

### 2.8. FFA Analysis

FFAs were extracted from the digesta using a liquid–liquid extraction procedure. Briefly, 500 μL of digesta was transferred into a 12 mL glass tube and mixed with 10 μL of an internal standard solution (10 μg/mL; FFA 16:0-d4). Two milliliters of dichloromethane was added, and the mixture was vortexed for 10 min. Subsequently, 2 mL dichloromethane and 1.6 mL ultrapure water were added, followed by centrifugation at 4000 rpm for 10 min. The lower organic phase was collected and evaporated under a nitrogen stream. The remaining aqueous phase was re-extracted twice with 2 mL dichloromethane (vortex 3 min, centrifuge 4000 rpm for 3 min). All organic fractions were combined and dried under nitrogen. The residue was reconstituted in 150 μL dichloromethane/methanol (1:1, *v*/*v*), passed through a 0.22 μm organic membrane filter, and subjected to UPLC-QTOF-MS analysis.

Chromatographic separation was performed on a Shimadzu LC-30AD liquid chromatography system (Shimadzu Corporation, Kyoto, Japan) coupled to a SCIEX TripleTOF 6600 mass spectrometer (SCIEX, Framingham, MA, USA). Separation was achieved on a Phenomenex Kinetex C18 column (100 × 2.1 mm, 2.6 μm; Phenomenex, Torrance, CA, USA). The injection volume was 3 μL, the flow rate was 0.4 mL/min, the column temperature was 60 °C, and the autosampler temperature was 4 °C. Mobile phase A was water/methanol/acetonitrile (1:1:1, *v*/*v*/*v*) containing 5 mM ammonium acetate, and mobile phase B was isopropanol/acetonitrile (5:1, *v*/*v*) containing 5 mM ammonium acetate. The gradient was 20–40% B from 0.5 to 1.5 min, 40–60% B from 1.5 to 2 min, 60–95% B from 2 to 10 min, held at 95% B for 2 min, and returned to 20% B at 13.1 min, followed by equilibration to 14 min.

Mass spectrometric detection was performed using electrospray ionization in negative mode over m/z 100–1200. The source parameters were: curtain gas, 35 psi; ion source gas 1, 50 psi; ion source gas 2, 50 psi; ion spray voltage, −4500 V; and source temperature, 600 °C. FFA identification was performed using MS-DIAL 4.9, PeakView, and MasterView. Semi-quantification was performed using FFA 16:0-d4 as the internal standard, with peak integration and calculation conducted in MultiQuant and an in-house macro-tool. FFA contents were expressed as μg/mg digesta.

### 2.9. Statistical Analysis

Data are presented as mean ± standard deviation (SD). The independent digestion experiment was used as the experimental unit for statistical analysis. Analytical replicates obtained from the same digesta were averaged before analysis and were not treated as independent experimental replicates. For datasets involving both sample type and digestion phase, two-way analysis of variance (ANOVA) was used to evaluate the effects of sample type, digestion phase, and their interaction. When significant effects were detected, Tukey’s post hoc test was applied for multiple comparisons. For endpoints analyzed within a single digestion phase, one-way ANOVA followed by Tukey’s post hoc test was used. Normality and homogeneity of variance were checked before ANOVA. Statistical significance was defined as *p* < 0.05. Statistical analyses were performed using GraphPad Prism 10, Origin 2024.

## 3. Results

### 3.1. Apparent Protein Digestibility

**Figure 1:**
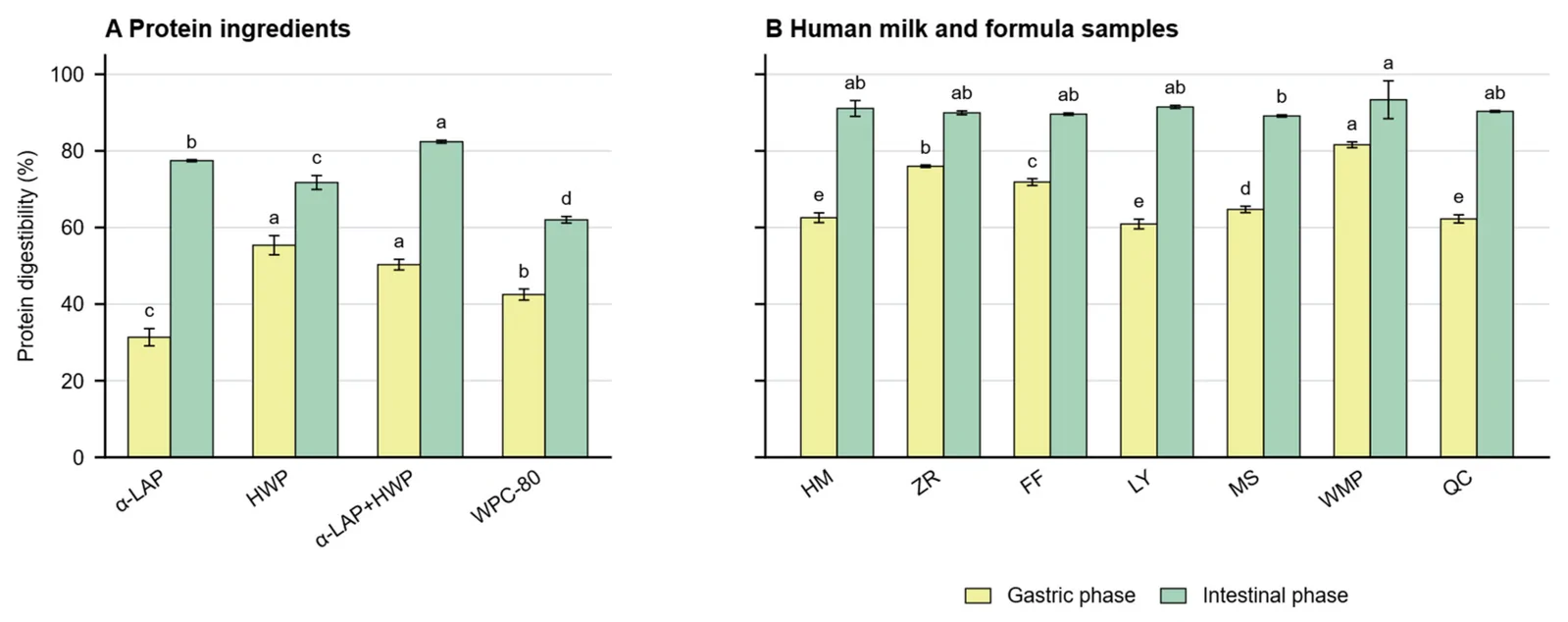
Apparent protein digestibility of protein ingredients, human milk, and infant formula samples during simulated gastrointestinal digestion. Protein digestibility of protein ingredients (**A**) and human milk/formula samples (**B**) after the gastric and intestinal phases of simulated digestion. Different lowercase letters indicate significant differences among samples within the same digestion phase. Data are presented as mean ± SD (*n* = 3 independent digestion experiments).

Significant differences in apparent protein digestibility were observed among the samples (*p* < 0.05), indicating that protein structure, compositional ratios, and matrix characteristics collectively influenced digestive behavior (Figure 1).

#### 3.1.1. Gastric Digestibility

Among the protein ingredients, HWP exhibited the highest gastric digestibility (55.37 ± 2.51%), followed by the α-LAP/HWP mixture (50.31 ± 1.39%), whereas α-LAP alone showed the lowest value (31.34 ± 2.25%). Overall, the protein ingredients showed lower gastric digestibility than the commercial dairy products and HM. Unlike whey-only ingredients, commercial products and HM contain caseins, which possess open and flexible structures that are rapidly cleaved under acidic gastric conditions. This pattern was further reflected in comparisons among HM, IFs, and WMP. WMP exhibited the highest gastric digestibility (81.59 ± 0.75%), significantly exceeding both HM (62.56 ± 1.27%) and all IFs (*p* < 0.05).

#### 3.1.2. Intestinal Digestibility

In the intestinal phase, the commercial dairy products and HM exhibited significantly higher digestibility than the protein ingredients (*p* < 0.05). Among the protein ingredients, WPC-80 showed the lowest intestinal digestibility (62.01 ± 0.86%).

Digestibility differences were also observed among the IFs, WMP, and HM, although these variations were less pronounced than those in the gastric phase. WMP exhibited the highest intestinal digestibility (93.36 ± 4.94%), whereas most IFs showed values comparable to HM (91.09 ± 2.06%), with no significant differences. IF MS showed the lowest intestinal digestibility within the IF group (89.13 ± 0.28%). These findings align with previous studies reporting that HM and IFs achieve similar total digestibility, with the primary divergence occurring during the gastric phase [33,34]. It should be noted that digestibility values in this section refer to apparent protein digestibility based on the decrease in residual TCA-precipitable protein fraction.

### 3.2. Protein Profiles

**Figure 2:**
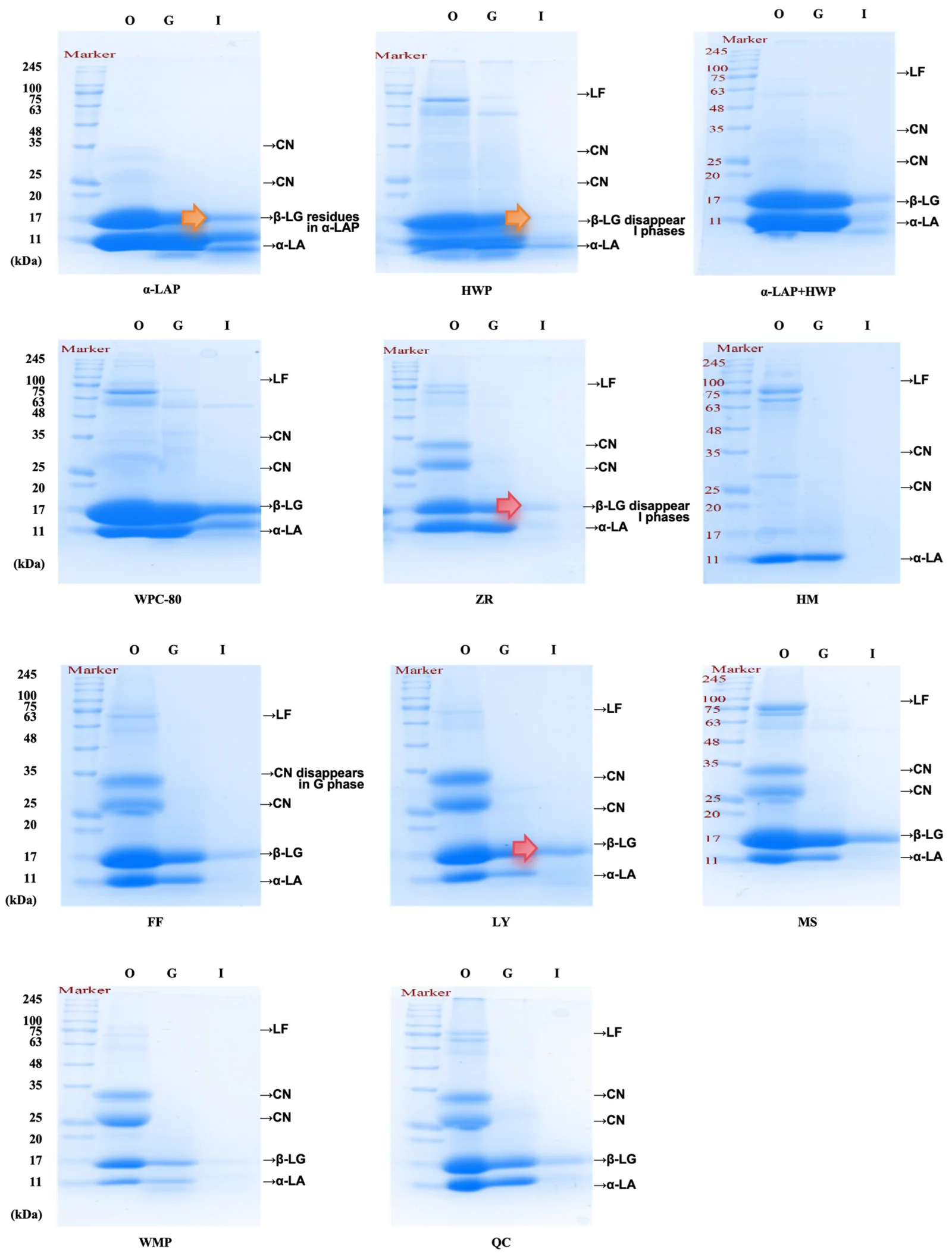
SDS–PAGE profiles of all samples at three digestion stages. O indicates undigested samples, G indicates samples after the gastric phase, and I indicates samples after the intestinal phase. Arrows indicate residual protein bands after digestion.

SDS-PAGE analysis revealed clear differences in protein degradation patterns among the samples across the undigested, gastric, and intestinal stages (Figure 2). In the undigested state, HM was characterized by bands corresponding to α-LA, LF, and serum albumin, while β-lactoglobulin (β-Lg) was absent, consistent with its natural protein composition and clearly distinguishing it from bovine milk-based IFs and WMP. 

Some IFs are formulated to increase the proportion of α-LA and reduce casein to better emulate the HM whey profile. Accordingly, α-LAP was used as a key biomimetic ingredient. The SDS–PAGE pattern of α-LAP displayed a noticeable amount of β-Lg, indicating the presence of residual whey proteins that remained after industrial purification; however, α-LA remained the dominant species. In addition, IFs fortified with lactoferrin exhibited stronger lactoferrin bands in the undigested stage, in line with their ingredient composition.

During the gastric phase, casein bands disappeared in all IF samples, whereas α-LA and β-Lg bands remained clearly detectable. In the intestinal phase, except for digestive enzymes, HM showed almost complete disappearance of protein bands, indicating extensive hydrolysis into low-molecular-weight peptides. In contrast, several IFs retained visible α-LA or β-Lg bands, suggesting that these whey proteins were not fully hydrolyzed under infant intestinal conditions.

### 3.3. Curd Formation and Flocculation Behavior

**Figure 3:**
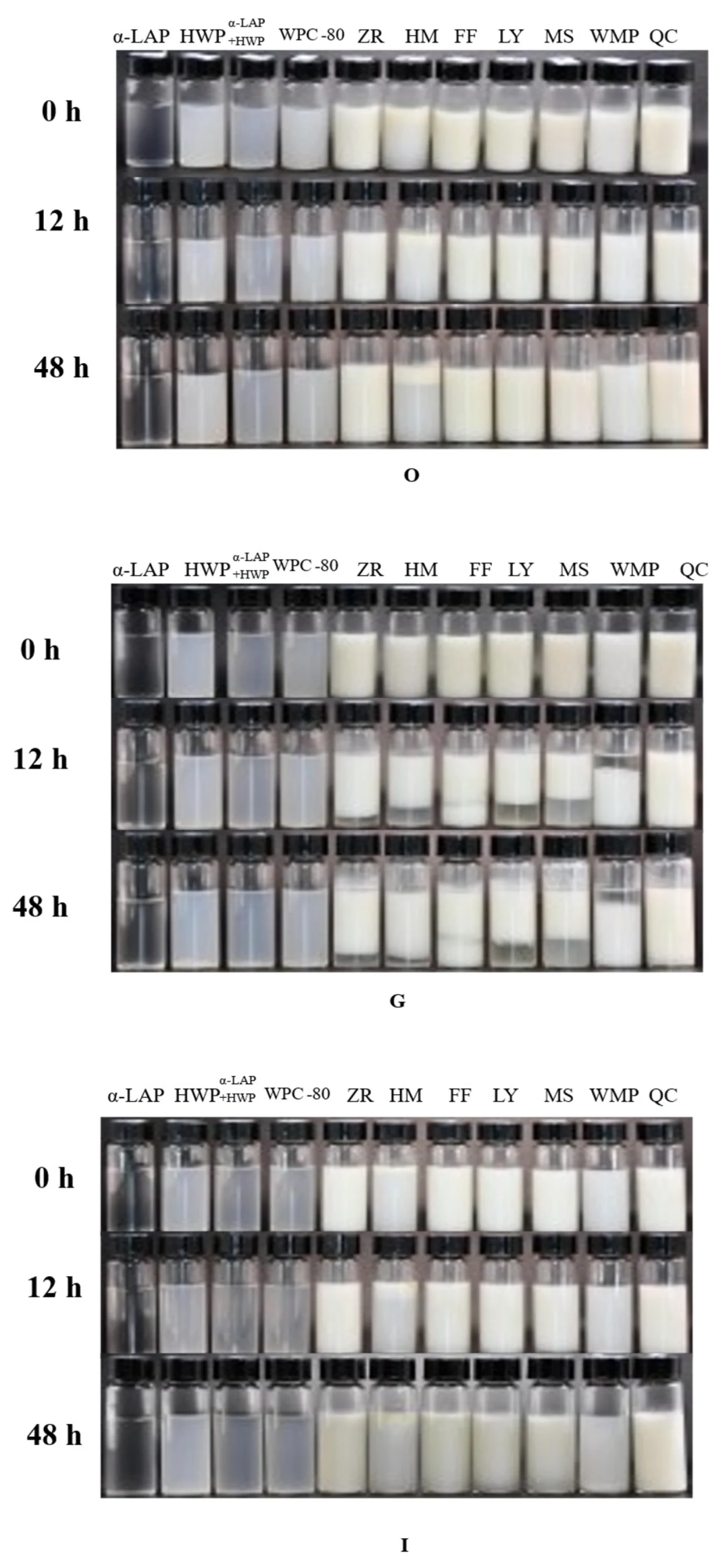
Curd formation and flocculation behavior of HM, IFs, WMP, and protein ingredient samples during simulated gastrointestinal digestion. O represents undigested samples, G represents gastric digestion, and I represents intestinal digestion.

To compare structural transformations among samples during gastrointestinal digestion, curd formation and flocculation behavior were evaluated immediately after sampling and after static settling for 12 and 48 h across the three digestion stages (Figure 3). At the time of immediate sampling, all systems appeared uniformly dispersed without visible aggregation phase separation, which was attributable to continuous stirring during the in vitro digestion process. Upon entering the intestinal phase, the overall appearance became lighter in color.

After 12 h of static settling, distinct curd morphologies were observed. Whey protein-based ingredients showed no visible sedimentation, indicating limited ability to form cohesive curd networks under gastric conditions. In contrast, both HM and IFs developed floating, low-density flocculated curds characterized by loose and porous structures. WMP formed dense, compact curds that settled at the bottom, producing a clear supernatant layer.

### 3.4. Particle Size

**Figure 4:**
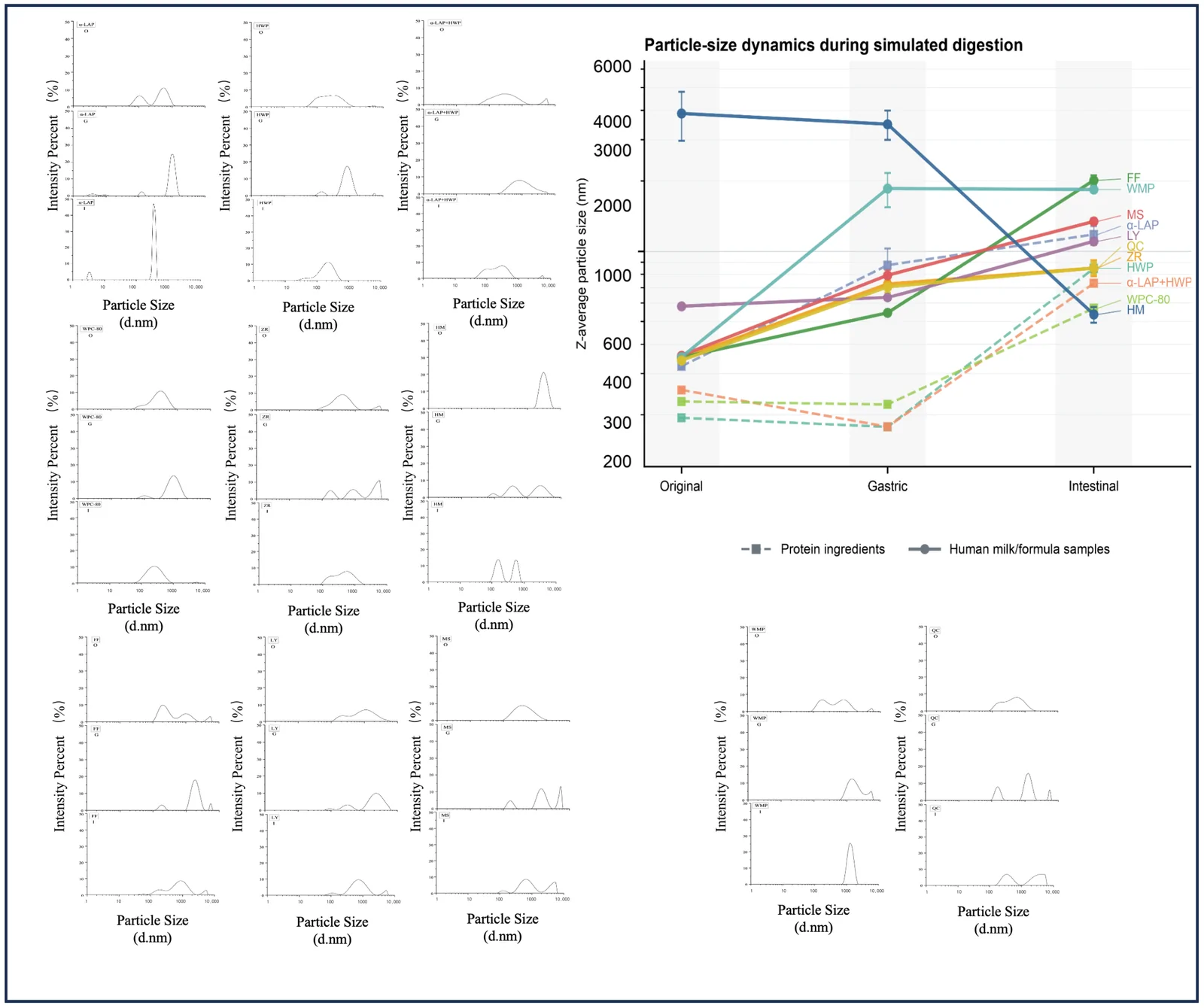
Particle-size dynamics of protein ingredients, human milk, and infant formula samples during simulated gastrointestinal digestion. Samples were measured in the original state and after the gastric and intestinal phases. Dashed lines with square markers indicate protein ingredients, whereas solid lines with circular markers indicate human milk and infant formula samples. The small, embedded panels show representative particle-size distribution profiles of the corresponding samples at different digestion stages (*n* = 3 independent digestion experiments).

The particle size changes of the samples during gastrointestinal digestion are shown in Figure 4. At the initial stage, IFs exhibited relatively uniform and significantly smaller particle sizes than HM.

During the gastric phase, the particle size of all samples increased significantly (*p* < 0.05), accompanied by the formation of curd structures. Notably, IFs exhibited a greater increase in particle size than HM, likely due to their higher casein proportion and denser, less flexible interfacial membranes, which are more susceptible to acid-driven aggregation.

Upon entry into the intestinal phase, the particle size of all samples decreased significantly (*p* < 0.05), reflecting progressive curd disintegration under the action of pancreatic proteases and bile salts. HM did not show the pronounced gastric-to-intestinal particle size reduction observed in IFs and WMP. It should be noted that the digesta were diluted with deionized water to meet the obscuration requirement for laser diffraction, which may have affected ionic strength and weak aggregates. Therefore, the particle-size data should be interpreted as comparative values under standardized measurement conditions.

### 3.5. Fatty Acid Composition

#### 3.5.1. Fatty Acid Profiles of Undigested Samples

**Figure 5:**
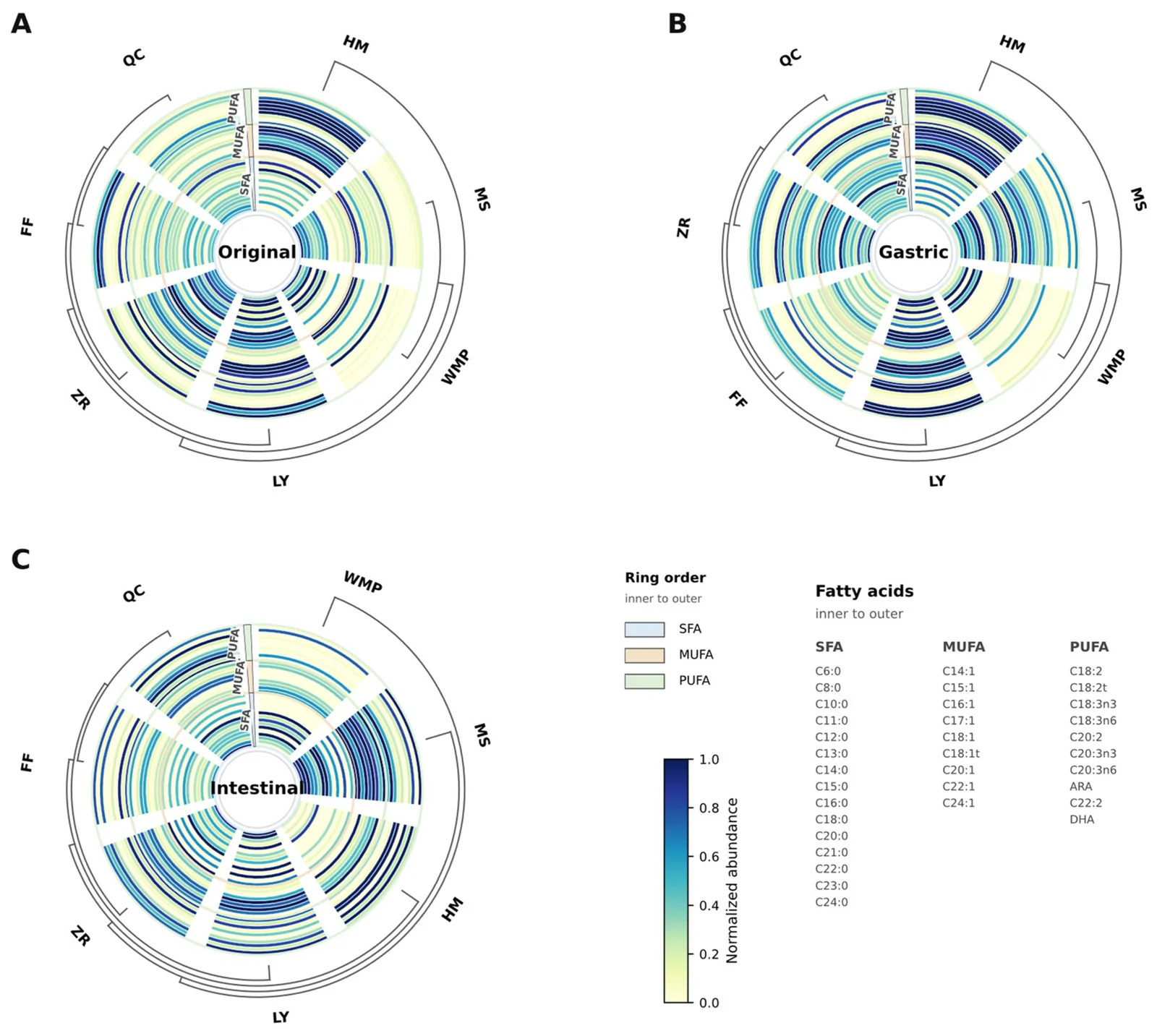
Stage-specific circular clustered heatmaps of fatty acid profiles during simulated infant digestion. (**A**) Original samples before digestion; (**B**) gastric digesta; (**C**) intestinal digesta. Sectors represent samples, and concentric rings represent individual fatty acids arranged from the inner to outer layers according to the order listed in the right-side legend: saturated fatty acids (SFAs), monounsaturated fatty acids (MUFAs), and polyunsaturated fatty acids (PUFAs). Color intensity indicates min–max normalized fatty acid abundance within each digestion stage (*n* = 3 independent digestion experiments).

Hierarchical clustering of the fatty acid profiles in undigested samples (Figure 5) revealed clear source-dependent differences among HM, IFs, and WMP. HM contained relatively higher levels of several polyunsaturated fatty acids (PUFAs), including C20:3n6, C20:2, C20:3n3, C16:1, and C18:3n6, while showing low levels of ruminant-derived fatty acids such as C18:2n6t, C15:0, C17:1, and C13:0. In contrast, WMP exhibited substantially higher proportions of odd-chain and trans fatty acids, presenting a characteristic ruminant fat fingerprint, consistent with its lipid fraction being predominantly derived from bovine milk fat [35,36]. Notably, MS clustered closely with WMP, indicating that its fatty acid profile was more similar to bovine-milk-derived lipids. This pattern aligns with publicly available ingredient information showing that MS incorporates dairy fat together with conventional vegetable oils but does not include OPO-based structured lipids.

Therefore, IFs are typically enriched with vegetable oils to provide adequate essential fatty acids and to improve overall lipid bioavailability. Formulas ZR, FF, and QC from the same manufacturer clustered together, indicating strong similarity in their lipid profiles. Ingredient information confirms that these formulas incorporate structured lipids such as OPO (sn-2 palmitate) together with vegetable oils.

#### 3.5.2. Fatty Acid Profiles After Gastric Digestion

Hierarchical clustering of the fatty acid profiles after gastric digestion is shown in Figure 5. Overall, the clustering structure remained similar to that observed in the undigested stage and continued to be driven primarily by the original lipid source and matrix characteristics. HM formed a distinct cluster; WMP remained closely grouped with MS; ZR, FF, and QC clustered together; and LY separated into an independent branch. These findings indicate that although lipolysis had begun in the gastric phase, the fatty acid patterns at this stage largely reflected the inherent lipid origin rather than digestion-induced transformation.

In HM, long-chain polyunsaturated fatty acids (e.g., C18:3n6, C20:2, C20:3n6, C20:3n3) remained dominant, consistent with its intrinsic PUFA-rich profile. WMP continued to exhibit enrichment in ruminant-characteristic fatty acids (e.g., C11:0, C13:0, C15:0), reflecting its bovine-milk-fat origin. Notably, the IFs produced by the same manufacturer (ZR, FF, and QC), which contain OPO structured lipids, remained tightly clustered after gastric digestion. This indicates a high level of consistency in their fatty acid release behavior and suggests that their lipid formulation systems remained stable throughout gastric digestion.

#### 3.5.3. Fatty Acid Composition During the Intestinal Phase

Hierarchical clustering of the fatty acid composition after intestinal digestion (Figure 5) revealed a markedly different clustering pattern compared with the undigested and gastric phases. The grouping structure previously dominated by fat source began to dissolve, and similarities among samples reflected their digestive behavior and lipolysis outcomes rather than their original lipid origin.

WMP remained clustered as a distinct group, characterized by relatively high proportions of ruminant-associated fatty acids such as C11:0, C13:0, and C18:0 (Figure 5). Cluster analysis of fatty acid categories (SFA, MUFA, PUFA) further confirmed that WMP contained higher SFA abundance and lower UFA levels than HM and IFs, consistent with its bovine-lipid origin.

In contrast, the distance between HM and several IFs (QC, FF, ZR, LY) decreased substantially during the intestinal phase, demonstrating a clear post-digestion convergence phenomenon. Notably, MS, which previously clustered closely with WMP in the undigested and gastric stages, shifted toward the IF cluster following intestinal digestion.

### 3.6. Released FFA Profile

**Figure 6:**
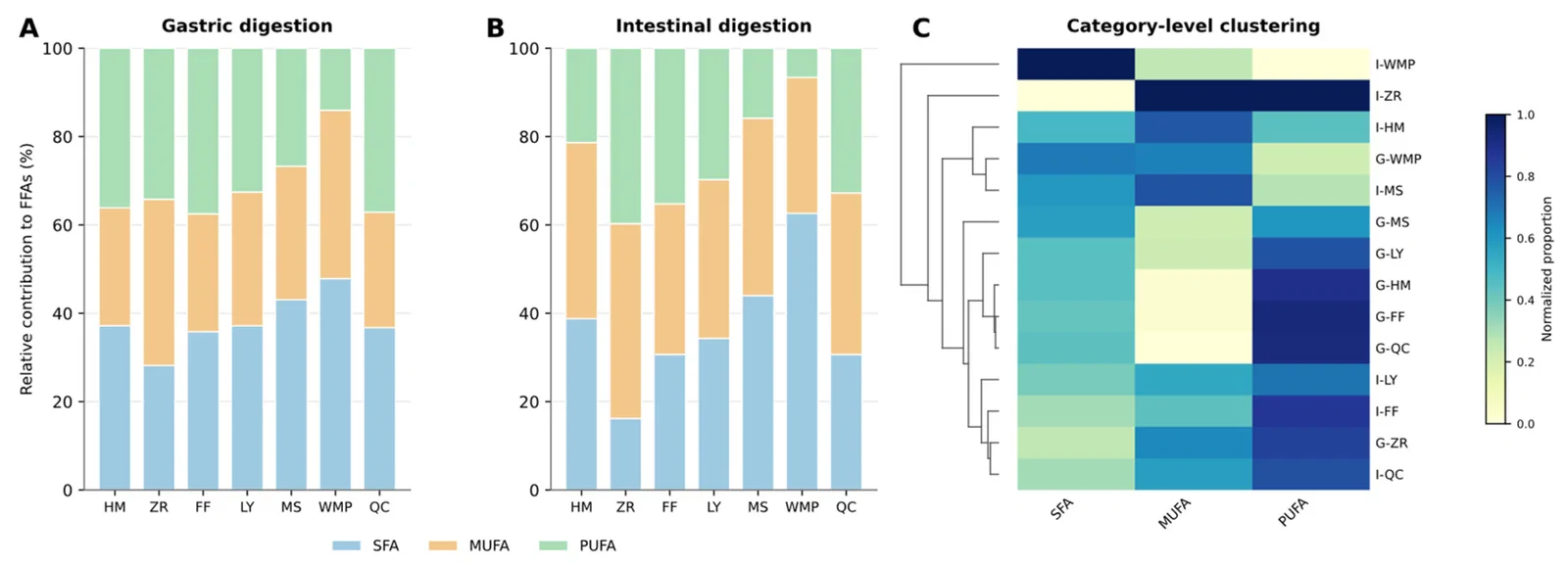
Relative distribution and clustering of saturated, monounsaturated, and polyunsaturated fatty acids in released free fatty acids during simulated digestion. (**A**) Relative distribution of SFA, MUFA, and PUFA in FFAs released after gastric digestion. (**B**) Relative distribution of SFA, MUFA, and PUFA in FFAs released after intestinal digestion. (**C**) Hierarchical clustering heatmap based on the relative proportions of SFA, MUFA, and PUFA in gastric and intestinal digesta. G, gastric digestion; I, intestinal digestion; HM, human milk; WMP, whole milk powder (*n* = 3 independent digestion experiments).

To compare the compositional characteristics of lipolysis products, the released FFAs were grouped into SFA, MUFA, and PUFA and expressed as relative proportions (Figure 6). After gastric digestion, all samples showed mixed contributions of SFA, MUFA, and PUFA, but the relative balance differed among milk systems. After intestinal digestion, the differences became more evident. HM showed a relatively high contribution of unsaturated fatty acids, whereas WMP was characterized by a higher SFA proportion among released FFAs. This pattern indicates that the lipid source and matrix structure influenced the compositional profile of FFAs generated during digestion.

Hierarchical clustering based on SFA, MUFA, and PUFA proportions further separated the samples according to their released FFA profiles (Figure 6C). The clustering results showed that intestinal digesta did not simply follow the same grouping pattern as gastric digesta, suggesting that intestinal lipolysis reshaped the relative distribution of released fatty acids. Several infant formulas showed FFA profiles that were closer to HM than to WMP, whereas WMP retained a more SFA-rich pattern. These results suggest that, beyond the total extent of lipid digestion, the relative composition of released FFAs provides additional information on the digestion-related similarity between infant formulas and HM.

### 3.7. Integrated Analysis of Formulation Features and Digestion-Related Similarity to HM

**Figure 7:**
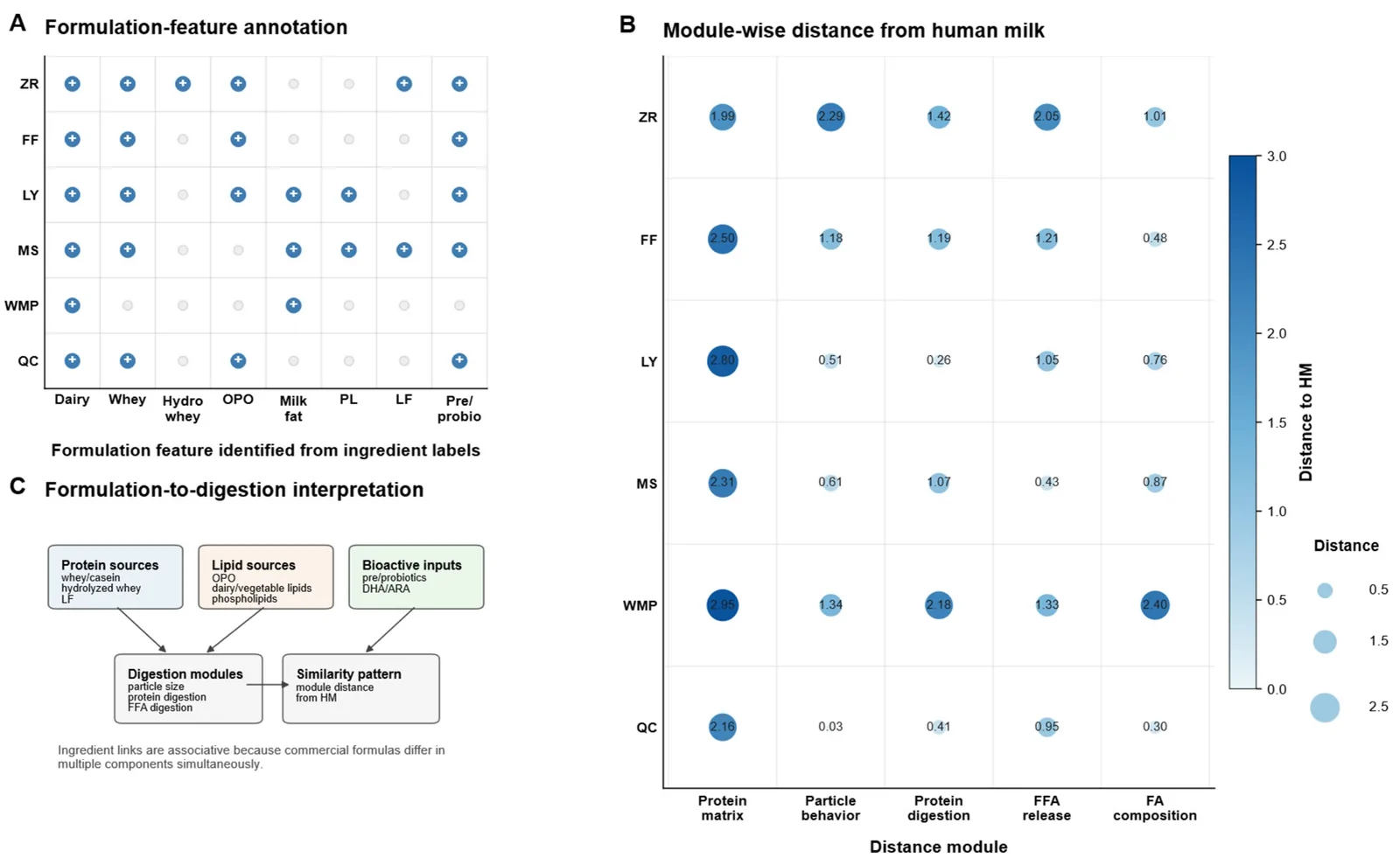
Integrated formulation–digestion similarity analysis of infant formulas relative to human milk. (**A**) Formulation-feature annotation based on ingredient-label information. Filled circles indicate the presence of the corresponding formulation feature. (**B**) Module-wise distance of each sample from HM. Bubble size and color intensity indicate the Euclidean distance from HM within each module; lower values indicate greater similarity to HM. (**C**) Schematic interpretation linking formulation inputs to digestion-related modules and HM-similarity patterns.

To further compare the digestion-related characteristics of the samples, formulation features were annotated from ingredient-label information and combined with module-wise distance analysis relative to HM (Figure 7). Five modules were included: protein matrix, particle behavior, protein digestibility, FFA release, and fatty acid composition. For each module, the Euclidean distance from HM was calculated after feature-wise z-score normalization. A lower distance indicates that the sample was closer to HM in the corresponding module.

The distance map showed that the samples differed across modules rather than following a single similarity pattern (Figure 7B). QC had the lowest overall mean distance from HM (0.77), followed by MS (1.06) and LY (1.08), while WMP showed the highest mean distance (2.04). At the module level, QC was closest to HM in particle behavior (0.03), protein digestibility (0.41), and fatty acid composition (0.30). Based on the ingredient-label annotation, QC contained whey ingredients, OPO, and pre/probiotic components.

MS showed a different pattern. Although its overall mean distance was not the lowest, it had the lowest distance in the FFA release module (0.43). MS contained milk fat/cream, phospholipids, lactoferrin, and pre/probiotic components, but did not contain OPO. WMP showed the largest overall distance from HM, especially in protein matrix, protein digestibility, and fatty acid composition. This result indicates that a bovine dairy matrix alone did not reproduce the digestion-related profile of HM. QC showed the closest overall profile to HM, mainly driven by particle behavior, protein digestion, and fatty acid composition, whereas MS was more similar to HM in the FFA release module.

## 4. Discussion

This study compared the gastrointestinal digestion behavior of HM, IFs, WMP, and key dairy protein ingredients using an infant static digestion model. The results show that digestion behavior was not determined by composition alone, but by the combined effects of protein structure, lipid source, processing-induced physical state, and digestion-induced structural transformation. Overall, the gastric phase preserved strong matrix-dependent differences, whereas the intestinal phase tended to attenuate some of these differences through proteolysis, bile salt-mediated interfacial displacement, re-emulsification, and lipolysis [29,32,33].

Protein digestion was strongly influenced by molecular conformation and processing-induced structural state. Among the protein ingredients, HWP showed higher gastric digestibility, which can be attributed to the presence of low-molecular-weight peptides generated by prior hydrolysis. In contrast, α-LAP showed lower gastric digestibility, consistent with the compact globular structure of α-lactalbumin and the limited accessibility of pepsin to buried cleavage sites [37,38,39]. WPC-80 also showed relatively low intestinal digestibility, which may be related to heat-associated denaturation and aggregation during ultrafiltration concentration and spray drying [40,41]. These findings indicate that protein digestibility depends not only on protein type, but also on the physical state generated during processing. The SDS-PAGE results support this interpretation. Casein bands disappeared rapidly during gastric digestion, consistent with the open and flexible conformation of caseins and their susceptibility to pepsin hydrolysis. In contrast, α-LA and β-Lg were more resistant, probably because their compact globular structures restrict enzyme access to cleavage sites [37,38,42]. The near-complete disappearance of α-LA and β-Lg bands in ZR further suggests that partial whey-protein hydrolysis improved enzyme accessibility and promoted more complete degradation during gastrointestinal digestion [14,43,44].

The different behaviors of HM, IFs, and WMP further highlight the role of casein structure and curd formation. WMP showed the highest gastric protein digestibility, probably because of its high casein proportion and the open, flexible structure of caseins under acidic conditions [42,45,46]. However, this high digestibility was accompanied by the formation of dense, bottom-set curds, which differed markedly from the loose and floating flocculated curds observed in HM and most IFs. This difference may be explained by the higher mineralization and calcium association of bovine casein micelles, which favor the formation of compact curd networks [47,48,49]. In comparison, HM casein micelles are less mineralized and more hydrated, producing softer curds with lower density [48,49]. Therefore, a higher extent of hydrolysis does not necessarily indicate a more HM-like digestion pattern. From a biological perspective, this distinction is important because infant digestion should not be interpreted simply as maximizing protein hydrolysis. A more HM-like pattern may involve coordinated gastric structuring and subsequent intestinal disassembly, allowing nutrients to be released progressively rather than being rapidly hydrolyzed within a compact curd matrix. In this context, the loose and floating curds observed in HM and most IFs may favor gradual enzyme access and transfer into the intestinal phase, whereas the dense bottom-set curds formed by WMP suggest a different gastric retention and release behavior. Therefore, apparent digestibility needs to be interpreted together with curd structure and particle behavior when evaluating the physiological relevance of infant formula digestion.

Consistently, HM showed a more balanced digestibility across gastric and intestinal phases, whereas WMP reached the highest total digestibility through a hydrolysis pattern that differed from the physiological digestion profile of HM, which is characterized by gradual nutrient release and interface-regulated proteolysis [33,50,51].

Particle-size evolution supported this interpretation. IFs initially showed smaller and more uniform droplets than HM, most likely due to high-pressure homogenization and spray drying [52,53]. HM, in contrast, contains larger native fat globules surrounded by an intact milk fat globule membrane, giving it a more complex and flexible interfacial structure [18,54,55]. During gastric digestion, acidification and pepsin activity promoted droplet aggregation and protein coagulation in all samples [56,57]. Lower pH can reduce electrostatic stability at the droplet surface, while pepsin-induced hydrolysis destabilizes interfacial proteins and facilitates coalescence and flocculation [53,58]. The subsequent decrease in particle size during intestinal digestion indicates that pancreatic enzymes and bile salts disrupted these aggregates and promoted re-emulsification, thereby increasing lipase accessibility to lipid droplets [59,60]. These structural changes also provide a link between protein and lipid digestion. During gastric digestion, protein coagulation and interfacial protein hydrolysis can alter droplet aggregation, thereby changing the surface area and accessibility of lipid droplets to digestive lipases. During intestinal digestion, bile salts and pancreatic enzymes may disrupt protein-stabilized aggregates and remodel the lipid interface, promoting re-emulsification and FFA release. Thus, particle-size evolution should be viewed not only as a physical change, but also as an indicator of protein–lipid co-digestion that connects curd formation, droplet accessibility, and lipolysis behavior.

The fatty acid results show that lipid source dominated the profiles before digestion and after gastric digestion. HM formed a distinct cluster, WMP retained a bovine milk fat signature, and several IFs with similar formulation strategies clustered together. These source-dependent differences are consistent with the known compositional gap between bovine milk fat and HM fat, particularly in essential fatty acids and ruminant-associated fatty acids [36,61,62]. Although bovine milk fat and HM share multiple fatty acid species, bovine milk fat alone does not provide sufficient linoleic acid and α-linolenic acid to meet infant formula compositional requirements; therefore, vegetable oils are commonly incorporated to achieve regulatory and nutritional targets [6,63].

The clustering of ZR, FF, and QC was consistent with their shared use of structured lipids such as OPO and vegetable oils. OPO can increase the proportion of palmitic acid esterified at the sn-2 position of triacylglycerols, thereby partially mimicking the stereospecific structure of HM fat [29,64]. However, the present results also show that lipid similarity cannot be explained by OPO alone, because formulas differed simultaneously in milk fat, vegetable oils, phospholipids, emulsified structure, and other ingredients.

After intestinal digestion, several IFs shifted closer to HM in fatty acid composition or released FFA profiles, suggesting a digestion-induced convergence. This convergence may be related to bile salt-mediated interfacial restructuring, pancreatic lipase activity, and the formation of mixed micelles [59,60,65]. The released FFA profile is also likely affected by the positional distribution of fatty acids within TAGs, because pancreatic lipase preferentially hydrolyzes fatty acids at the sn-1 and sn-3 positions. The distribution of SFAs and UFAs within TAGs is not random but is influenced by species, lactation stage, and diet [61,66,67]. In HM, unsaturated fatty acids are relatively enriched at these positions, which may explain the higher contribution of UFAs among intestinal FFAs [68,69]. This interpretation is consistent with previous infant digestion studies showing that intestinal digestion can alter the relative composition of released FFAs [70]. Nevertheless, this explanation remains inferential because the present study did not directly measure TAG sn-position distribution or lipid interfacial composition.

The integrated formulation-digestion analysis further indicates that HM similarity is multidimensional. QC showed lower distances from HM in particle behavior, protein digestibility, and fatty acid composition, whereas MS was closer to HM in the FFA release module. This pattern suggests that different formulation strategies may reproduce different aspects of HM digestion rather than uniformly improving all digestion-related properties. Importantly, these results should not be interpreted as a ranking of product quality. Because commercial formulas differ in multiple ingredients at the same time, including protein sources, whey ingredients, structured lipids, milk fat, phospholipids, lactoferrin, and pre/probiotic components, the present study cannot assign causality to any single ingredient. This limitation is particularly relevant because milk fat globule structure, phospholipid composition, and bioactive protein addition can all influence digestion-related behavior [10,18,23].

Several limitations should also be considered. First, the static infant digestion model cannot fully reproduce dynamic gastric emptying, continuous pH changes, time-dependent enzyme secretion, intestinal absorption, or individual infant variability. Therefore, the present results should be interpreted as standardized comparative endpoints rather than as a complete representation of in vivo digestion kinetics. Second, Rhizopus oryzae lipase was used to provide gastric lipase activity in the present model. Although this allowed gastric lipolysis to be included under acidic infant digestion conditions, this enzyme should not be considered equivalent to human gastric lipase because its substrate specificity and activity profile may differ. In addition, FFA concentrations were obtained using a single-internal-standard-based semi-quantitative method; thus, these data were used to compare released FFA profiles among samples and digestion phases, but not as fully validated absolute quantification of lipolysis extent.

Overall, this study shows that mimicking HM digestion requires attention to dynamic digestive behavior rather than static composition alone. Protein conformation, curd structure, fat droplet interface, fatty acid composition, and intestinal re-emulsification together determine how milk systems are digested under infant gastrointestinal conditions. These findings provide a basis for understanding protein–lipid co-digestion in early life and may help guide the development of infant formulas with digestion behaviors closer to those of HM. Future studies should directly characterize TAG positional distribution, lipid interfacial composition, and digestion-derived peptides to further clarify the mechanisms underlying these differences.

## Data Availability

The original contributions presented in this study are included in the article/Supplementary Materials. Further inquiries can be directed to the corresponding authors.

## Supplementary Materials

The following supporting information can be downloaded at: https://www.mdpi.com/article/10.3390/foods15172999/s1, Table S1: Initial Compositional Information.

## Abbreviations

The following abbreviations are used in this manuscript:

HM	Human milk
IF	Infant formula
WMP	Whole milk powder
α-LA	α-Lactalbumin
α-LAP	α-Lactalbumin powder
β-CN	β-Casein
β-Lg	β-Lactoglobulin
LF	Lactoferrin
HWP	Hydrolyzed whey protein
WPC-80	Whey protein concentrate 80
OPO	1,3-Dioleoyl-2-palmitoylglycerol
FFA	Free fatty acid
SFA	Saturated fatty acid
MUFA	Monounsaturated fatty acid
PUFA	Polyunsaturated fatty acid
UFA	Unsaturated fatty acid
TAG	Triacylglycerol
MFGM	Milk fat globule membrane
SGF	Simulated gastric fluid
SIF	Simulated intestinal fluid
BCA	Bicinchoninic acid
SDS-PAGE	Sodium dodecyl sulfate–polyacrylamide gel electrophoresis
FAME	Fatty acid methyl ester
GC	Gas chromatography
FID	Flame ionization detector
PL	Phospholipids
DHA	Docosahexaenoic acid
ARA	Arachidonic acid
